# Practitioners’ Views on Enabling People With Dementia to Remain in Their Homes During and After Crisis

**DOI:** 10.1177/07334648221118557

**Published:** 2022-08-25

**Authors:** Marcus Redley, Fiona Poland, Donna Maria Coleston-Shields, Miriam Stanyon, Jennifer Yates, Amy Streater, Martin Orrell

**Affiliations:** 1 6106 School of Health Sciences, University of East Anglia, Norwich, UK; 2 2152 Department of Psychiatry, University of Cambridge, Cambridge, UK; 3 6123 Institute of Mental Health, University of Nottingham, Nottingham, UK; 4 Research and Development, Northeast London NHS Foundation Trust, Ilford, London, UK

**Keywords:** dementia, community, qualitative methods, health services, mental health

## Abstract

One way of supporting people living with dementia is assisting them to live in their homes (as opposed to being admitted to hospital or other facility) and providing them with a specialist service that responds to crises. This makes it important to understand how best to organize such crisis response services. This study examines practitioners’ actions to reduce inpatient admissions among this population. Through interviews with healthcare practitioners, we find that practitioners negotiate a complex intersection between (1) what constitutes a *crisis* in relation to the patient and/or the carer, (2) the demands of building a working relationship with both the patient and their family carers, and (3) ensuring effective communications with social services responsible for long-term community support. Findings suggest that policies aimed at reducing admissions should be based on a model of care that more closely maps practitioners’ relational and bio-medical work in these services.

**What this paper adds**

• Practitioners negotiate a complex intersection between what constitutes a *crisis*, working relationships with patients and carers, and liaison with social services
• Policies for reducing admissions need a model of care that closely maps practitioners’ relational and bio-medical approaches

**Applications of study findings**

• Crisis services for people with dementia need a national model of care appropriate for this patient population.
• The service should be multi-disciplinary and recognize both the clinical and relational needs of such patients and the vital role played by family carers.
• This model should also reduce inpatient admissions by enabling people with dementia to live in their homes for longer whilst supporting their wellbeing.

Dementia is a significant threat to global health (World Health Organization, 2012), the leading cause of disability and dependency amongst older people (Alzheimer’s Disease International, 2013), and a condition principally managed through the provision of personal assistance (Prince et al., 2013). Hence, policies for the care and treatment of people living with dementia are geared toward supporting them to live in their homes for as long as possible (Department of Health, 2009, 2015) and reducing the need for inpatient hospital admission often through specialist clinical teams that respond to crises in community settings. However, the policies do not clearly define what constitutes a *crisis* (Vroomen et al., 2013) and clinicians, family carers, and people with dementia can hold differing views on what a crisis is (Hopkinson et al., 2020). Moreover, while there is an emerging body of literature seeking to characterize crises in dementia care (e.g., Hopkinson et al., 2020), little is known about what practical actions specialist crisis teams take to reduce inpatient admissions and what model of care captures the work of these practitioners, and the policy environment they operate within.

## Background

In the UK, the policy environment leading to the emergence of *crisis response teams* began over 20 years ago. In 1999, aiming to actualize care in the community and reduce the cost of inpatient admissions, mental health trusts were directed to introduce 24-hour crisis response teams (Department of Health, 1999) that would help support people experiencing a mental health crisis to stay in their homes. While initially conceived for people of working age with a functional mental illness, this reform gradually evolved to include people living with dementia. In addition, a separate program of reforms, known as “personalization,” (Department of Health, 2007) sought to give people receiving health and social care services greater control over how their care and support was planned and delivered. Under the *Care Act,* for instance, practitioners are required to place the wellbeing of those needing support, their needs and goals, at the center of their care and support plans (Department of Health, 2022) which also applies where a person lacks capacity. When making substitute decisions, the *Mental Capacity Act* requires practitioners to consider their patients’ views and feelings (Mental Capacity Act, 2007). In addition, both the national dementia strategy, *Living Well with Dementia* (Department of Health, 2009), and the *Prime Minister’s Challenge on Dementia 2020* (Department of Health, 2015) explicitly promote the empowerment of service users with dementia. Empowerment was intended to both enhance wellbeing and cut costs on the basis that people generally prefer to remain in their homes and that homecare should be cheaper than hospital or nursing home admission (Department of Health, 2007, 2010). In a related effort to improve financial efficiency, the government actively promoted greater integration between healthcare provided by NHS trusts and social care funded by local authorities. The assumption was that better integration would lead to people with health and social care needs being able to continue living in their homes for longer (Department of Health, 2013, 2017). However, there was no blueprint for how crisis response services should be organized or managed, and this responsibility lay with the 191 local commissioning groups and 60 mental health trusts comprising the National Health Service. Consequently, there remained considerable variation in how crisis response services for older people were organized, with some supporting adults of all ages experiencing a mental health crisis and others supporting either only older adults or people with a diagnosis of dementia (Streater et al., 2017). These variations, plus local demographic and geographic differences, reflected the limited guidance for, or evidence of, the best ways to organize effective crisis response services (Streater et al., 2017). Nevertheless, it is not generally the severity of dementia that predicts hospital admission, but other factors including multiple health conditions, polypharmacy, and dependency (Shepherd et al., 2019), alongside domestic instability (Perlman et al., 2018). Thus, in order to help inform guidance on how best to organize crisis response services for people with dementia, this study aimed to investigate the practical steps practitioners in specialist crisis teams follow to reduce inpatient admissions.

## Method

### Design

To ascertain the actions taken to reduce inpatient admissions, we interviewed a sample of practitioners working in crisis response teams, analyzing the resulting data using interpretative phenomenological analysis (Smith & Shinebourne, 2012).

### Selection and Research Instrumentation

The participants of this study were selected from five NHS community mental health trusts from across England concerned with developing the quality and effectiveness of specialist crisis response teams. These trusts were involved in a larger study AQUEDUCT (Broome et al., 2020). Of the five teams involved in the present study, four serviced older adults, including those with dementia, while one was a specialist service exclusively for people living with dementia. Sites were selected to include urban and rural settings. The participants were selected pragmatically (including based on who was available for interview) and all had the experience of responding to crises involving people with dementia. They included 12 nurses (including two clinical team leaders), one psychiatrist, one occupational therapist (also a clinical team leader), and one healthcare assistant. The interviews were conducted by J. Yates and M. Stanyon, two female post-doctoral researchers with degrees in psychology. Each interview began with the interviewers explaining the aim of the research. The interviews themselves comprised eight questions covering four broad topics: instances where participants considered patient outcomes to have been successful, examples of less successful outcomes, circumstances supportive of good practice, and views on how good practice might be developed (see Supplement Appendix 1).

### Data Collection and Ethical Consideration

All interviews were conducted one-to-one at the participants’ places of work and lasted a maximum of 1 hr. Prior to the interviews, each participant provided informed consent in writing. The interviews were transcribed verbatim. Ethical approval was obtained from the NHS Health Research Authority.

### Data Explication

The analysis was a two-stage process. First, each interview topic was considered an interpretative theme (Cicourel, 1964) with participants’ answers summarized question by question. These summaries, allowing a degree of immersion in the data, became the basis for identifying new or emergent themes (Braun & Clarke, 2006). The research team validated both the summaries and identified three themes that seemed central to understanding how the participants perceived their role and the actions they took (see Table 1) and reflected how team members (i) define or understand what a crisis is, (ii) divide their work into making clinical interventions and establishing relations with patients and carers, and (iii) work with other health and social care services. These three themes link participants’ understanding of their role in managing inpatient admissions (Smith & Shinebourne, 2012). Since our analysis took participants’ accounts at face value (Silverman, 2017), no attempt was made to explore how participants rhetorically constructed their answers (Potter & Wetherell, 1987) or how those answers might have been influenced by interviewer–interviewee interaction (Mishler, 1991). We followed the COREQ guidelines for reporting qualitative research (Tong et al., 2007).

**Table 1.** Data Analysis.

Stage 1		Stage 2	
Initial questions and initial themes		Data immersion and emergent themes	
Questions	Themes	Data immersion	Emergent themes
Q2: Can you think of a case where you were really satisfied with how your team provided care? Please explain what happened?	Satisfactory outcomes for patients	Immersion in the data summarizing answers to each question identification of emergent themes	
Q3: Can you think of a case where you were less happy with the care that was provided by the team or when things didn’t work out in the way you had wished? Can you explain what happened?	Less successful outcomes		Team members definition or understanding crises
Q6: How do you feel when things go well? Less well?			Team members describe their work in terms of both clinical interventions and establishing relations with patients and carers
Q4 What helps you provide “ideal practice”?	Circumstances supportive of good practice		
Q5: Can you think of things that get in the way of providing this “ideal practice”?			Team members describe working with other health and social care services
Q7 How do you think current practice can be improved?	How good practice might be developed		
Q1: Personal details: Job role, time in post	Background data		
Q8: Further comments?			

## Findings

Generally, the clinical practitioners interviewed described dementia as a chronic progressive condition for which medical interventions can only delay an inevitable cognitive deterioration. They described many dementia patients as suffering physical health problems often related to physical frailty or specific conditions such as urinary tract infections, which can further impair their cognition. Only a minority of patients were also described as depressed and/or exhibiting challenging behaviors. The practitioner interviews included accounts of reviewing patients’ medication, screening for physical health conditions, and identifying the possible causes of any behaviors that might lead to physical harm or reckless use of financial resources. Additionally, many participants reported offering reassurance and guidance to family carers. Ultimately, however, their accounts served to justify decisions (Potter & Wetherell, 1987) about whether a person should continue to live in their home, perhaps with additional support from other services, or change accommodation. The latter option could involve moving to a community residential home, or an older adults’ mental health unit in the case of severe challenging behavior. When reporting judgments of this kind, in line with government policy, the practitioners clearly preferred care within the patients’ home. Admission as an inpatient, also in line with government policy, was seen as regrettable, except in cases where a patient’s behavior went beyond a carer’s ability to cope with it. Underlining that they were simply providing a response to a crisis, participants neither perceived themselves as providing the kind of long-term care necessary for a patient to remain in their home once the crisis had passed, nor as responsible for arranging changes of accommodation (although they might make recommendations about support required). However, because of the slow pace at which some commissioners and providers of social care services worked, the participants reported instances where patients remained within a crisis response service despite the immediate crisis being over.

### Theme 1: Different Understandings of Crises

When participants had experienced one or more service reorganizations, they were able to comment on how the characteristics of a crisis response differed according to different service models. For instance, where new eligibility criteria meant that clinical teams were now caring for a broader range of patients, they reported that some clinicians lacked the necessary skills for working with older people. Participants suggested that colleagues lacking these skills were more likely to respond paternalistically, over-estimating risks, or, conversely, more readily accept patients’ refusal of care and support. Moreover, they reported that age-related physical health problems and signs of dementia could be overlooked. However, they perceived generic adult teams as more likely to offer a 24-hour service compared to specialist services, which were open only to people with dementia. The participants also reported that clinical practitioners who regularly worked with working-age adults with functional mental health problems viewed crises primarily from a suicidal risk perspective. In contrast, practitioners working mainly with older adults understood crises as situations in which, for example, a patient wanders out of their home in a confused state (see Excerpt 1) or when family carers struggle to cope with the patient’s challenging behavior (see Excerpt 2).

#### Excerpt 1

…and it’s trying to, you know, [when] you [as a working-age colleague] have a young person who wants to jump off a bridge and kill themselves. To the working-age people, that’s a crisis. If you have somebody who is wandering away from their home because of their cognitive problems, that’s a crisis, but they don’t see the comparison […]. [02–07 Nurse]

#### Excerpt 2

…so sometimes, you know, it might be as simple as providing a short-term sedative to take the edge off, particularly for when people are aggressive, or it could just be a case of sitting down and supporting the carer, going through a few things that they might be doing. [02–05 Nurse]

In other words, these practitioners defined a crisis as pertaining to providing personal assistance rather than as something grounded solely in a person’s mental state. The implications of this distinction included when practitioners reflected that involvement with a crisis response team was short term, allowing only limited opportunities for engaging with and supporting family carers. These constraints on involvement led some participants to conclude that their service was not particularly sensitive to the realities of living with dementia (see Excerpt 3).

#### Excerpt 3

I think we are pretty good at person-centered [care], but I think [there is a] desire to rush out [terminate involvement]; I mean, one of my colleagues the other day said, “We should just go whoosh, and out”. Imagine trying to find a model of care that [goes] “whoosh and then out”. […] So [its clear] we’ve got different models in our heads. And I’ve always struggled with this twenty-one- or twenty-two-days business [as the ideal length of service involvement], which was probably suggested by people who don’t know about dementia. [01–08 Nurse]

### Theme 2: Clinical Interventions and Establishing Relations With Patients and Carers

When describing their work, some practitioners divided patients with dementia into two groups. One group comprised younger male patients aged 50–70 years, whose dementia had a rapid onset. These men, who were physically strong, were often described as behaving violently toward their frailer wives and partners. It was for this group that the participants deemed admission to an inpatient older adult mental health unit most appropriate. The other group, exemplified through references to the proverbial “little old lady,” whilst not exclusively female, were represented as an older and physically frail population. Although they might exhibit challenging behaviors, the practitioners did not consider them a likely risk to family members who were providing personal assistance. The practitioners did not consider admitting them to an inpatient older adult mental health unit, where patients were consistently described as noisy and aggressive, appropriate for them. Practitioners preferred to see these more frail patients supported in the community, either in their homes or in community residential homes. It was this latter group of people and, more specifically, those living in their homes, that practitioners seemed most eager to discuss when describing their successes.

For the purpose of this analysis, participants’ accounts of working with this population have been conceptualized as involving two distinct but related areas of clinical competence: the *bio-medical/clinical* and the *relational*. While this distinction was not always clear-cut in the interviews, the participants differentiated between those tasks associated with assessing a person’s mental and physical health, and those focused on building and sustaining relations with the patient, and perhaps more importantly, the family members providing the personal assistance enabling the patient to remain in their home.

Regarding bio-medical/clinical tasks, these could involve reviewing medication, arranging clinical tests, or making referrals to other clinical services. Where interventions involved medication, team members with prescriber status (medical doctors and some nurses) reported that, because of their involvement, patients benefitted from having their physical health and medications more speedily and frequently reviewed (see Excerpt 4).

#### Excerpt 4

[…] sometimes we can go in and see somebody twice a day. That could be to prompt medication [intake], monitor medication side effects, prompt food and drink [intake], make sure that they are up out of bed and kind of starting to function, if that is something that they have been struggling with. So, we can go in and do that piece of work with them and then hopefully kind of get things moving a bit quicker than liaising with GP’s […]. [05–04 Nurse]

Practitioners might also advise family carers on how they might avoid “triggering” challenging behavior by adapting the home environment and/or changing the way they interact with the person for which they provide care. Where they considered a carer’s need for support to be acute because the patient could not be left alone for any length of time, a crisis response might also involve arranging for a health care assistant or nurse to make daily visits. These visits allowed family carers time for their own personal care while the health care assistant or nurse provided personal care to the patient: helping the person with dementia to get out of bed and get dressed, ensuring adequate nutrition and hydration, and ensuring that medications were taken. In addition, the practitioners reported that assisting family carers in this way enabled them to model good practice for the benefit of family carers. Some practitioners also reported that they would initiate contact with other services, particularly a local authority’s adult social care service, to arrange respite care and/or a statutory needs assessment, under the Care Act (2014), for the dementia patient and/or their carer.

With respect to the relational aspect of their involvement, the participants described such things as bringing a dispassionate viewpoint to a situation; listening empathetically to carers’ concerns and worries and offering practical suggestions on how best to care for the person with dementia (see Excerpt 5).

#### Excerpt 5

Quite often [there is] reassurance [in] having somebody like myself turn up, talk through things, put things into perspective, and point out perhaps a few changes that the carer could make in how they are providing care for their husband, wife, or whomever. I always found that actually, that went a [long] way in resolving what you would call a crisis. [02–05 Nurse]

Just as important, and in keeping with the idea that in this patient population, it is often the person providing care who is in a crisis, was offering reassurance to carers. The participants reported that working with a carer included educating them about dementia, especially to help them understand that the condition is progressive and incurable, as well as helping them come to a consensus over what is in the patient’s best interests. This might mean instilling the belief that they can cope and continue caring for their spouse or relative (see Excerpt 6).

#### Excerpt 6

I remember my first encounter with a wife [caring for her husband]. I knocked and she was alone in the house and it was just a matter of “how are you getting on?” “how is the situation for you?” We had a lengthy chat for about an hour and a half […] Sometimes [they need] that person […] who empathizes with [them], because they understand how [they] feel, so it [feel] like [they] are alone. So, the whole process with the family, they really trusted us. And whatever we gave them in terms of intervention, sometimes not medication, sometimes liaising with the social worker for them, calling them, was a very lengthy process. At the end of the day, they [the family] were aware that things will decline, but they were happy that we sort of took them there slowly. [01–09 Support Worker]

Winning the family carers’ trust was not automatic. The participants observed that gaining trust could be hindered or facilitated by factors such as length and frequency of appointments, the ability to respond flexibly to carers’ needs when making appointments, shift patterns and working hours that mitigated against the continuity of care, and timely responses from other services when their input was requested. They reported that it was harder to win carers’ trust and cooperation when they held hostile views toward institutionalized care, whether in community residential homes or in an inpatient older adults’ unit. Crucial to strengthening the relational aspect of a team’s involvement was informing both the person with dementia and those caring for them that, as the team was responding to a crisis, their involvement would be time-limited and focused on resolving the crisis that led to their involvement.

The participants’ accounts of their practice varied in the prominence given to either the *bio-medical/clinical* or *relational* aspects of their involvement. Practitioners with prescriber status or operating within generic adult services tended to emphasize the clinical aspects of their involvement, whilst those without prescriber status and/or working within specialist dementia services emphasized the significance of their relational work. This emphasis on relational work led one participant to question whether she and her colleagues really did anything of substance because building and sustaining relations with people with dementia and carers was hard to describe objectively. Despite relational work being a key feature of some participants’ conception of a crisis response service, participants rarely referred to the *Mental Capacity Act* and their legal responsibility for ensuring that people with dementia are involved in decisions about their care and management.

### Theme 3: Working With Other Health and Social Care Services

When describing working relations with other services, practitioners described swift onward referral to another service as a successful outcome. Whether the person with dementia remained in their home, with or without additional support or adaptions, or moved to a community residential home, was not straightforward to accomplish. Practitioners described how poor communication between members of the team and staff in other health services and social care services could delay discharge. This was most apparent in descriptions where two or more services were supporting the same dementia patient but not coordinating their efforts. Participants attributed poor communication to changes in personnel, staff taking annual leave, and service reorganization. In addition, they sometimes portrayed the local authority staff as lacking the experience necessary for working with older adults. In one instance, this led to avoidable delay in arranging an appointment with an occupational therapist, as local authority staff were apparently unwilling to work with a person who had a “complex mental health condition.” They reported that delays like this undermined trust with family carers. While our interviews were not written to elicit participants’ views on the funding of social care, the subject frequently arose in their accounts of working with social care services. We were told, for instance, about people assessed as having sufficient financial assets to fund their own care and support (so-called “self-funders”), who did not purchase support for themselves at the advised level. Additionally, local authority funding panels did not always approve support packages at the recommended level. The consequences of such a decision were described by one practitioner as a placement breakdown and required the submission of a new funding application to convince the local authority funding panel to purchase support at the recommended level. When talking about funding arrangements, participants did not always appear fully informed about their legal basis; some referred to agreements made under Section 75 of the *National Health Services Act* (in which local authorities and NHS trusts could pool resources), which were abolished in 2012 with the introduction of Clinical Commissioning Groups (Health and Social Care Act, 2012). A few participants spoke about the wider socio-economic context, including cuts in funding or services. For example, reductions in inpatient beds could mean that people admitted to older adult units were unable to be placed in or near their community, and patients were being supported to stay in their homes at a time when community services were being cut back (see Excerpt 7).

#### Excerpt 7

I guess it always comes down to funding at the end of the day with a lot of things, doesn’t it? Social services have had to cut a lot of their funding, so we can’t always get the amount of support that we used to get. Like with day services, people going into day services for care […]. There isn’t that sitting service anymore either, where families can go out [while paid support workers look after the person with dementia]; that provision has been taken away […] and that is quite difficult for some people. So, they manage at home for a lot longer, but then it gets to a crisis point. [05–04 Nurse]

## Discussion

Practitioners’ efforts to reduce inpatient admissions lay at an intersection between (i) understanding crises as either problems inherent to patients or problems residing with a carer struggling to cope; (ii) the dynamic between practitioners focusing on patients’ mental and physical health needs, or building and sustaining interpersonal relations with family carers; and (iii) communication and understanding among social services colleagues responsible for supporting people to remain in their community. Despite the limitations of focusing on participants’ own accounts of their practice, this research does illuminate practitioners’ efforts to reduce inpatient admissions.

### Different Understandings of Crises

In common with other studies (see Hopkinson et al., 2020 for a recent review), we did not find a consistent definition of what constitutes a crisis in dementia. Some services used a model of care devised for working-age adults with functional mental illnesses, where crises were frequently understood in terms of suicide risk, while some participants defined crises in terms of family members no longer being able or willing to provide personal assistance to an older adult with dementia. Services aiming to support people living with dementia to remain living in their homes will require a model of care that, while not focused exclusively on the needs of carers, recognizes this relational dimension and its demanding nature for carers.

### Clinical Interventions and Establishing Relations With Patients and Carers

Our findings revealed the importance of both bio-medical/clinical interventions and building relations with family carers (Toot et al., 2013). However, in practice, individual practitioners often appeared to focus on just one role or the other. Practitioners with prescriber status tended to emphasize the bio-medical clinical aspects of their involvement, seeing patients as benefiting from having their physical health and medications more speedily and more frequently reviewed. Consequently, the extent to which a crisis response team could effectively meet both needs may depend on the distribution of skills within a team. While many practitioners valued supporting and enabling family carers to continue providing personal assistance, they often did so by describing their own individual practice, rather than identifying how they worked as part of a multi-disciplinary team. Our findings also indicated that younger male patients who behaved violently toward their wives and partners could be at increased risk of inpatient admission (Wharton & Ford, 2014). Whether such admissions were unavoidable or attributable to community services that lacked the required clinical or relational skills or resources is a moot point requiring further study.

### Working With Other Health and Social Care Services

When commenting on other services, practitioners noted poor communication and failures of coordination (Jacobsohn et al., 2019), which they attributed to regular changes in personnel, holiday-linked absences, and service reorganizations. The staff in local authorities were often regarded as lacking the necessary skills to work with older adults. That said, some practitioners had little understanding of how social care was funded, which suggested that they might also not understand the wider context in which their specialist service operated. Findings suggest that coordination between health and social care services must improve further (NHS, 2019).

## Conclusion

Reducing impatient admissions for persons living with dementia is a national policy objective (Department of Health, 2009). However, variations in how crisis response teams were organized meant there was insufficient robust evidence to determine how best to organize services (Streater et al., 2017).

### Theoretical Contributions

Having examined the actions practitioners took to reduce inpatient admissions, it is apparent that crisis services need to develop a national model of care appropriate to this patient population. Such a model would recognize both the clinical and relational needs of such patients (Ryvicker et al., 2021) and the vital role played by family carers (Griffin et al., 2020). As providing a crisis response service requires effective communication between healthcare and social services, a comprehensive model of care needs to recognize and support multi-agency working.

#### Practical Contributions

The autonomy of people with impaired capacity (Lord et al., 2015) and relational needs must be respected. Equally important are the needs of those providing personal assistance (Livingston et al., 2014) as are approaches to build-up their resilience (Sonola et al., 2013). With respect to challenging behavior, it is important to recognize that such behaviors can place a person at increased risk of inpatient admission.

### Future Directions

Further research is required to develop a national model of care for people with dementia and their carers during a crisis. Such a model would enable the identification, development, and sharing of best practices among practitioners in various agencies supporting people with dementia to remain in their homes during and after a crisis.

## Supplemental Material

Supplemental Material – Practitioners’ Views on Enabling People With Dementia to Remain in Their Homes During and After Crisis Click here for additional data file. Supplemental Material for Practitioners’ Views on Enabling People With Dementia to Remain in Their Homes During and After Crisis by Marcus Redley, Fiona Poland, Donna Maria Coleston-Shields, Miriam Stanyon, Jennifer Yates, Amy Streater, and Martin Orrell in Journal of Applied Gerontology
