# Supplementary material for: Practitioners’ Views on Enabling People With Dementia to Remain in Their Homes During and After Crisis
Source: J Appl Gerontol. 2022 Aug 25;41(12):2549–56. doi: 10.1177/07334648221118557 (PMC9669725; doi:10.1177/07334648221118557)
Supplement: Supplemental Material – Practitioners’ Views on Enabling People With Dementia to Remain in Their Homes During and After Crisis [file sj-pdf-1-jag-10.1177_07334648221118557.pdf]

Appendix 1

Prompts used to interview members of clinical team with responsibility for responding to crises

Personal details: job role, time in post

Can you think of a case where you were really satisfied with how your team provided care? Please explain what happened?

Can you think of a case where you were less happy with the care that was provided by the team or when things didn't work out in the way you had wished? Can you explain what happened?

What helps you provide "ideal practice"?

Can you think of things that get in the way of providing this "ideal practice"?

How do you feel when things go less well?

How do you think the current practice can be improved?

Further comments?

Comment [A1]: For improved readability, consider revising this as "Prompts used to interview clinical team members who provide care during crises."

Comment [A2]: Would "…when things do not go exactly as planned" better suited here?
